# Promoting Stair Climbing as an Exercise Routine among Healthy Older Adults Attending a Community-Based Physical Activity Program

**DOI:** 10.3390/sports7010023

**Published:** 2019-01-18

**Authors:** Nobuko Hongu, Mieko Shimada, Rieko Miyake, Yusuke Nakajima, Ichirou Nakajima, Yutaka Yoshitake

**Affiliations:** 1 Department of Nutritional Sciences, The University of Arizona, Tucson, AZ 85721-0038, USA; 2 Chiba Prefectural University of Health Sciences, Chiba 261-0014, Japan; mieko.shimada@cpuhs.ac.jp (M.S.); rieko.miyake@cpuhs.ac.jp (R.M.); 3 Comprehensive Welfare, Urawa University, Saitama 336-0974, Japan; nakajima@urawa.ac.jp; 4 Department of Physical Education, International Budo University, Chiba 299-5295, Japan; in-nakaj@budo-u.ac.jp; 5 National Institute of Fitness & Sports in Kanoya, Kagoshima 891-2311, Japan; yositake@nifs-k.ac.jp

**Keywords:** aging, stair-climbing, community-based program, physical activity, walking, pedometer

## Abstract

Stair climbing provides a feasible opportunity for increasing physical activity (PA) in daily living. The purpose of this study was to examine the daily walking and stair-climbing steps among healthy older adults (age: 74.0 ± 4.9 years; Body Mass Index (BMI): 22.3 ± 2.5 kg/m^2^). Participants (34 females and 15 males) attended a weekly 6-month community-based PA program. During the entire program period, daily walking and stair-climbing steps were recorded using a pedometer (Omron, HJA-403C, Kyoto, Japan). Before and after the 6-month program, height, body weight and leg muscle strength were assessed. After the 6-month program, the mean walking and stair-climbing steps in both women and men increased significantly (*p* ≤ 0.01). Daily stair-climbing steps increased about 36 steps in women and 47 steps in men. At the end of 6 months, only male participants had significant correlation between the number of stair steps and leg muscle strength (r = 0.428, *p* = 0.037). This study reported that healthy older adults attending the community-based PA program had regular stair-climbing steps during daily living. Promoting stair climbing as an exercise routine was feasible to increase their walking and stair-climbing steps.

## 1. Introduction

Aging-induced physiological changes such as declining strength, endurance, balance and cognitive decline [1] adversely affect the activities of daily living [2]. It is well documented that regular physical activity (PA) helps older adults improve their health, maintain independent living, and enhances their overall quality of life [3,4]. However, PA levels are low and decrease throughout aging in older adults [5,6]. Thus, older adults should be encouraged to maintain or increase regular PA levels [1,7]. In Japan 26.6% of the total population is 65 years old or older, ranking as the world’s oldest population [8]. It is projected that 40.1% of the total population will be 65 years old or older in 2050 [8]. More than 50% of older adults in Japan are not engaged in any PA [9]. Japanese health professionals are adopting the Ministry of Health Promotion Movement in the 21st Century (“Health Japan 21”) project, which promotes PA for the citizens of all ages in Japan, particularly targeting increasing the number of steps [10,11] and improving the ability to perform daily living activities [12].

Taking stairs is a frequently available form of PA that requires no special training or equipment. Stair climbing uses 8–10 times the energy requirements of the resting state, which makes stair climbing a vigorous daily PA [13,14,15]. The health benefits of regular stair climbing have been reported, including increased aerobic capacity [16,17], improved lipid profiles [18,19], and fitness and body composition [20]. Regular stair use was associated with lower risk of stroke in men who climbed 20–34 floors per week [21], and reduced risk of lung cancer [22]. Stair climbing for a short time decreased blood glucose levels in people with type 2 diabetes [23].

Recommending regular stair use is a great public health initiative to promote and to remain physically active. Numerous interventions have been conducted using various approaches, including motivational posters, signs [24,25], videos [26], environmental changes [27] and PA programming [28]. Many interventions promoted stair use settings at worksites [11,29], shopping malls and train stations [30]. A recent systematic review demonstrated the effectiveness of stair use and stair climbing interventions in various public settings [31]. However, these interventions were limited to office workers and younger adults. In one study, older men and women stair climbed 16–40 floors weekly for 8 weeks, resulting in significantly improved resting and exercise heart rates, and improved balance and perceived exertion. The authors showed that regular stair climbing can be done by older adults and can limit aging-induced physiological decline [17].

Popular walking programs use a pedometer to encourage older adults to be more physically active. However, the number of steps recommended for physically active or sedentary older adults to increase climbing up and down stairs as a part of an exercise routine is not known. No studies have examined the numbers of actual daily stair-climbing steps in healthy older adults. Therefore, the aim of the study was to examine the numbers of daily walking and stair-climbing steps among healthy older adults who were attending a weekly community PA program for 6 months. This study also seeks to examine whether the walking and stair-climbing steps are affected by the age, gender, leg muscle strength, and attending a community-based PA program. We hypothesized that promoting stair climbing as an exercise routine at a weekly community-based PA program increases the number of walking steps including stair-climbing steps among healthy older adults.

## 2. Materials and Methods

### 2.1. Participants and Study Design

The study participants were recruited from local community centers and residential communities. They live in the northwestern parts of Chiba Prefecture, Japan. The inclusion criteria were as follows: (a) age 65 years or older, (b) walk/move independently, (c) able to walk with gait patterns necessary to permit adequate pedometer reading, (d) wear a pedometer (wear clothing that permits placing a pedometer), and (e) have not been restricted from PA by a physician or a nurse practitioner. Participants were excluded if they had chronic neuromuscular or cardiopulmonary pathologies or terminal cancer. At the initial meeting, all participants (n = 74, mean age, 73.2 ± 5.0 years) read and signed an informed consent form. The study protocol was developed in accordance with the Declaration of Helsinki and was approved by the Research Ethics Committee of the Chiba Prefectural University of Health Sciences, Chiba, Japan (#2015-06).

### 2.2. Measurements

Sociodemographic and health-status variables: After participants provided written consent, sociodemographic variables (age, gender, city of residence) were collected. BMI was calculated from measured height and weight. Self-reported general health perception was measured using the Medical Outcomes Study 36-item short-form health survey (SF-36) in Japanese. The SF-36 contains 36 items measuring health and well-being. In this study, we reported the score of physical functioning —how difficult it was to climb several flights of stairs (answers: Yes, limited a lot (very difficult); Yes, limited a little (a little difficult); No, not limited at all (not difficult at all)). The Japanese version of the SF-36 has been validated for Japanese subjects [32].

Muscle strength: To assess muscle strength of upper limbs and hip abduction and adduction, Hip Power II (Model T.K.K. 3368, Takei, Scientific Instrument, Niigata, Japan) was used. Participants were seated with hips and knees at a 90° angle. They were instructed to keep the trunk straight, sitting as still as possible and gripping their chair throughout the test. They were instructed to abduct or adduct the hip. Hip Power II was held between the knees to measure adductor muscles strength. With attached belts holding legs, abductor muscles strength was measured. The train gauge load cell detects the force and displays the maximum value in kilogram (kg) per body weight in kg, while participants attempted to abduct or adduct the hip.

Physical activity: PA was assessed using the Omron HJ-403C, electronic pedometer (Omron Health Care, Kyoto, Japan) for the entire 6 months. Reliability of Omron HJ models in assessing steps under free-living/independent conditions have been established with adult populations [33,34]. The Omron HJ-403C pedometer detects the number of stair-climbing steps. Accuracy of counting steps during stair climbing was examined by one of the authors of this study (Shimada). HJ-403C pedometer stores walking steps for seven-days. It was automatically reset to zero daily at 12:00 midnight. Research assistants in the study helped each participant with the accurate placement of the pedometer on the waistline, clipped to a belt or clothing. Participants were asked to wear the pedometers during all waking hours except when bathing or swimming. When participants attended the weekly physical activity program, the research assistants downloaded the daily walking and stair-climbing steps from each participant’s pedometer into a computer for later analysis.

### 2.3. Intervention

**Figure 1:**
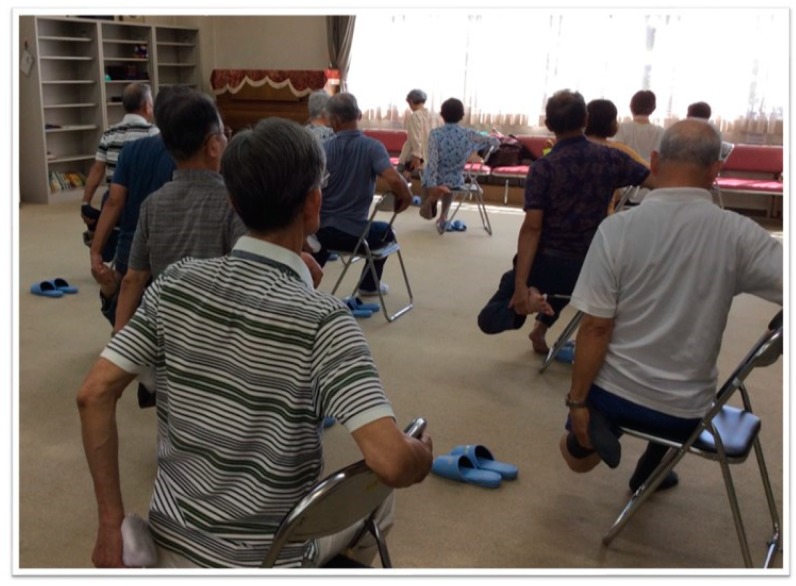
Session of community-based PA program, stretching.

The intervention program consisted of 1-h sessions held at a community center once per week for 6 months. A session consisted of a brief presentation on health-related tips such as increasing daily steps, overcoming barriers of PA, and an announcement of community PA events (first 2–3 min), warm-up activities (10–15 min), main exercise activities (30–35 min) and cool-down activities (10–15 min). Main exercise activities included balance and muscle-strengthening training, which included standing on one leg with eyes open, squats and lunges and finishing with stretches (Figure 1). All sessions were carried out by a trained fitness professional/instructor. During the weekly intervention program, the instructor encouraged participants to have stair climbing as their daily PA routine and have at least 150 minutes per week of total PA throughout the week [5]. The instructor explained the benefits of leg strengthening, as well as safely using stairs at home and outside of the home. The instructor listened to the participants and discussed their daily PA and routine stair use during the weekly sessions. The sessions were expected to develop the participants’ self-efficacy (belief that they could accomplish the goal) in doing PA, and build peer support for regular PA. The team of health professionals, physical fitness experts, nursing faculty members, and students from the research Universities were trained to support and network with participants during the program.

### 2.4. Statistical Analysis

Means, standard deviations, percentages and ranges were used to describe personal data, stair-climbing steps, walking steps, knee strength and SF-36 scores. A paired *t*-test was conducted to examine the changes in stair-climbing steps, walking steps, SF-36 scores, and knee strength before and after the intervention program. Spearman correlation coefficient was calculated to assess the relationship between stair climbing step counts and knee strength in both sexes. Statistical significance was set at *p* < 0.05 for all tests. All analyses were preformed using JMP Pro14.1.0 for Windows (SAS Inc., Cary, NC, USA).

## 3. Results

Of 74 study participants who completed baseline testing, 25 dropped out before completing the 6-month measurements, leaving a final number of 49 (34 females and 15 males), who we used for the analysis in this study. The reasons for dropout included illness, difficulty in traveling, and lack of continued interest. There were no significant differences in age, gender, BMI, and baseline daily step counts between participants who completed the study and those who dropped out (*p* > 0.05, data not shown). All participants were Japanese. The mean age ± SD was 75.1 ± 4.8 years in male and 72.4 ± 4.9 years in female participants. The age range was 64–85 years. The mean BMI for the total participants was 22.0 ± 3.0, with all participants having BMI below 25 and no participants having BMI of 30 or greater. There were no significant differences between male and female participants in terms of age and BMI, but there were significant differences in height and body weight (Table 1). The participants in this study used stairs during daily life activity. According to the results of physical functioning (SF-36), 76% of the participants reported stair use was not difficult at all, 20% of participants reported a little difficulty, and remaining 2 participants (4%) reported very difficult in stair climbing.

At baseline, both male and female participants recorded an average of more than 5000 steps daily; 8120 ± 3769 steps in male and 6493 ± 3705 steps in female participants. The range of daily steps in this group was 1239–19,292. Between the baseline and last sessions of the intervention at 6 months, the paired *t*-tests demonstrated significant improvement in the number of steps (i.e., walking and stair climbing) in both male and female participants (*p* < 0.05). After the 6-month program, female participants had significantly increased their daily walking steps compared to male participants (717 steps vs. 219 average steps per day). The average numbers of increased stair-climbing steps were 36 steps in females and 47 steps in male per day, which account about 0.2% of the total numbers of walking steps in both male and female participants (Table 2). During the 6-month intervention, no participants reported injurious falls, and the number of SF-36 (self-reported difficulty in climbing several flights of stairs) did not change in either male and female participants. Also, the BMI did not change. This indicates that the intensity and duration of PA are good enough for older adults to maintain their stamina and feelings of well-being.

**Figure 2:**
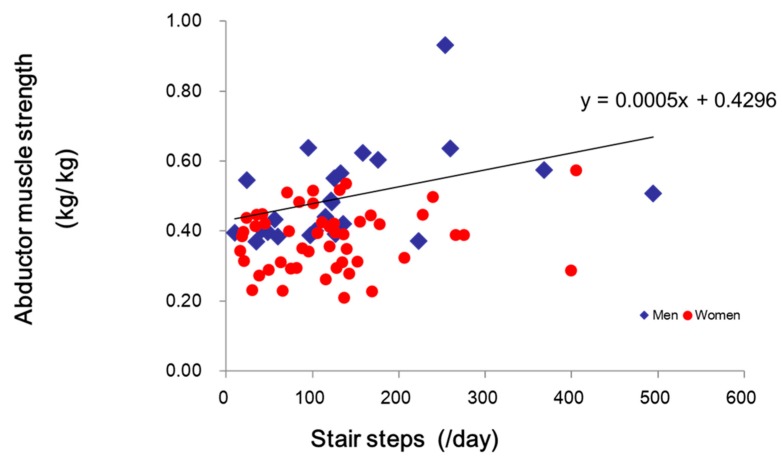
Daily stair steps and abductor leg muscle strength.

Muscle strength (i.e., knee extensor, abductor, abductor) did not change significantly in either group after the completion of the intervention program (Table 2). There was significant correlation between the abductor muscle strength and number of stair steps at 6 months only in male participants (*p* = 0.037) (Figure 2).

## 4. Discussion

The purpose of this study was to examine daily walking and stair-climbing steps using pedometers in older adults who were attending a community-based PA program, and determine if age, gender, leg muscle strength, and promotion of stair climbing by fitness professionals/community program instructors had any additional impact on their daily walking steps. To the best of our knowledge, this is the first study to show the number of stair-climbing steps during daily life activities in healthy older adults. Our findings have important implications for community PA program providers and older adult participants. Stair climbing is one of the essential functional activities for maintaining independence of daily living among older adults. However, little is known about the actual number of walking and stair-climbing steps, and limitations in climbing stairs by older adults [35]. The greatest contribution of this study was its evidence supporting a community-based PA program for increasing the number of daily stair-climbing steps among older adults. Findings from this study indicate that male and female participants significantly increased their walking and stair-climbing steps from baseline to 6-months, and the abductor muscle strength was significantly associated with numbers of walking and stair-climbing steps at 6 months only in male participants.

In this study, the average daily walking steps at baseline was 7301 steps, which falls in the ranges of steps (2000–9000 steps per day) reported in the review of pedometer-based PA interventions [36]. The baseline average daily walking steps in this study was similar to those found in studies conducted in similarly aged healthy older adults in the UK, the US, and Japan: 6509 steps (UK, Gale et al. [37]), 6443 steps (UK, Harris et al. [38]), 7721 steps (US, Kullgren et al. [39]), 5235 steps (US, Strath et al. [40]), and 7600 steps (Japan, Shephard et al. [41]). At the end of the 6-month PA program, both male and female participants significantly increased their daily walking steps. Female participants increased their daily walking steps by 717 steps, which represents an 11% increase from baseline. Male participants also increased by 212 steps over baseline, which represents 2.7%. All participants in this study negotiated stairs routinely in their homes or local community. They increased the numbers of stair-climbing steps by 23~25% from baseline to 6 months. At the end of the 6-month program, the average number of stair-climbing steps was 172 steps per day. According to other studies reporting stair-climbing speeds (1.1–1.7 steps per second) among older adults [42], the participants in this study may spend about 3–5 minutes per day stair climbing. The total of 172 steps represents about 5–6 floors ascending and descending the stairs (at 16 steps per level) in a typical building or parking garage [17]. 68% of male and 18% of female participants in this study had achieved a public health target of 8000 steps per day, which is the recommendation for older adults by Ewald et al. [43]. Although the intraindividual changes in number of both walking and stair-climbing steps were very small, with huge interindividual differences, it is important to note that these older participants in this study found their own ways to increase their steps throughout their regular daily life, while they were attending a weekly, 1-h community-based PA program.

The leg muscle strength did not change in either male or female participants who participated in the community-based PA program, even though they increased their walking and stair-climbing steps at the end of the 6-month program. As we mentioned in the methods section (Intervention), the instructor of this PA program delivered traditional, low-intensity balance training, and no machine-based muscle strength training was included in the program. This could be a limitation for increasing leg muscle strength in the participants of this study. Future efforts might be directed at creating more effective training programs for increasing leg muscle strength.

There are several considerations to keep in mind when interpreting our results. The simplicity and low cost of stair climbing encourages the formation of community groups with older adults to do PA, and this has the potential for the implementation of our study results at other community organizations, such as senior centers, retirement communities, and the Young Men’s Christian Association (YMCA). Recent systematic review and meta-analysis reported that exercise, particularly individualized types of exercise for older adults, are associated with lower risk of injurious falls compared with usual care [44]. However, one might argue that stair climbing as exercise may increase the risk of injuries in some older adults, because those people who are sufficiently mobile to use stairs are more likely to have a fall than sedentary older adults who do not use stairs. The ability to participate in stair climbing is affected by age-related changes, such as loss of muscle strength and balance, changes in visual conditions, cognitive decline, multiple medications, and presence of lower extremity pain [45,46]. Therefore, future studies are needed to better understand sensory-motor mechanisms and to identify older adults at high risk of falls in order to intervene in these risks and reduce stair climbing falls and injuries. Community-based programs may include enhancing awareness of injurious falls and promoting behavior changes to reduce risky choices while using stairs, thus improving safety and stair-gait performance.

This study has several limitations. First, because of the study design, the older adults who signed up to participate in this study were not blinded, and there is no control (non-exercise) group in this study. Thus, we do not know to what degree, if at all, the community-based PA program activities might have influenced the improvement in walking and stair-climbing steps, or if the participants in this study may have been simply motivated to be physically active. The second limitation is the small sample size of the study (n = 49). In addition, all participants in the present study were Japanese, recruited from community centers. All of them were functionally independent. Thus, generalizations should not be made. Third, the participants themselves were another limiting factor. This study was conducted in a real-world setting, meaning it was in our local community. However, recruiting approaches in a local community may have resulted in including participants with higher motivation to participate in the study. The older adults in this community willing to help University research projects, bringing about a Hawthorne effect, knowing that they would be meeting with researchers to report on their PA. They may have had additional motivation to increase or maintain their PA during the study period. Fourth, there are several limitations of using pedometers to measure PA. A pedometer is a simple and readily available instrument for both assessment and motivational purposes. The limitations of using pedometers include the fact that it only measures ambulatory activity—walking—and does not record cycling, swimming, or upper body work. Pedometers are less accurate for very slow walking [47], and will undercount if the pedometer is tilted off axis [43]. We used an Omron pedometer (HJ-403C), which has been found to be reliable during horizontal walking and stair ascending and descending. Ayabe et al. [48] reported that Omron pedometers could assess the number of steps within a ±5% error margin during stair ascending and descending at a stepping rate of 80–120 steps per minute in young men. Lastly, there is a lack of process data to determine what may have been associated with the program effects, thus leaving unknown which aspects of the community program were the most important. To be more precise, a larger randomized controlled trial is warranted to re-examine the results of the present study and explore the mechanisms.

In conclusion, the present study showed that healthy older adults had regular stair climbing during daily living. Participants in this study increased the daily walking and stair-climbing steps, which can simply be due to increasing motivation themselves while they were attending the weekly community-based PA program. Fitness professionals’ encouragement of daily stair climbing as part of an exercise routine may help motivate participants to increase their daily walking and stair climbing. In older adults, regular stair climbing might be a promising way to increase PA and maintain their independence during their daily lives. We consider that the findings from this study will be helpful for planning a fulfilling PA program delivered by fitness professionals.

## Figures and Tables

**Table 1:** Baseline characteristics of the study participants.

Variable		Male (n = 15)	Female (n = 34)
Age (years)		75.1 ± 4.8	72.4 ± 4.9
Weight (kg)		62.9 ± 8.77 *	50.5 ± 6.5 *
Height (cm)		166.3 ± 6.49 *	152.0 ± 4.9 *
BMI (kg/m^2^)		22.7 ± 2.21	21.9 ± 2.8
SF-36 (climbing several flight of stairs)	Yes, limited a lot	1	1
(n, number of people)	Yes, limited a little	3	7
	No not limited at all	11	26

Values are in mean ± SD or prevalence (%). BMI: body mass index. * *p* < 0.01.

**Table 2:** Changes in BMI, SF-36, daily steps and muscle strength between baseline and 6 months.

Variable	Male		Female	
	Baseline	6 months	Baseline	6 months
BMI (kg/m^2^)	22.7 ± 2.2	21.9 ± 2.2	21.9 ± 2.8	22.1 ± 3.3
SF-36 (climbing several flights of stairs				
Yes, limited a lot (n)	1	1	1	0
Yes, limited a little (n)	3	3	7	6
No, not limited at all (n)	11	11	26	28
Daily steps	-	-	-	-
Walking	8120 ± 3769	8339 ± 3207 *	6493 ± 3705	7210 ± 2441 *
Stair climbing	141 ± 113	188 ± 194 *	120 ± 86	156 ± 77 *
Muscle Strength	-	-	-	-
Adductor (kg/kg)	0.39 ± 0.11	0.41 ± 0.08	0.32 ± 0.09	0.32 ± 0.10
Abductor (kg/kg)	0.50 ± 0.13	0.50 ± 0.10	0.38 ± 0.09	0.40 ± 0.09

Values are in mean ± SD. * *p* < 0.05.
